# Long-term survival in venous thromboembolic disease: rivaroxaban vs. warfarin – propensity score matching study

**DOI:** 10.1186/s40360-023-00712-8

**Published:** 2023-12-13

**Authors:** Estefan Ramos-Isaza, Eduardo Tuta-Quintero, Alirio Bastidas-Goyes, Diana Diaz-Quijano, Carolina Aponte-Murcia, Julian Espitia-Angel, Daniel Pinto-Beltran, Johan Rincón-Hernández, Juan Sánchez-Cuellar, Jesus Pérez-Bueno, Luis F. Giraldo-Cadavid

**Affiliations:** 1 Medicine at Facultad de Medicina, Universidad de La Sabana, Universidad de La Sabana, Km 7, Autonorte de Bogota, Chía, Cundinamarca 250001 Colombia; 2 Clinica Universidad de La Sabana, Chía, Colombia; 3 Chief of the Interventional Pulmonology Service, Facultad de Medicina, Fundacion Neumologica Colombiana, Universidad de La Sabana, Bogotá, Colombia

**Keywords:** Rivaroxaban, Warfarin, Survival, Venous thromboembolic disease

## Abstract

**Background:**

Venous thromboembolic disease (VTE) is characterized by obstruction of venous blood flow by a thrombus. Survival data, frequency of disease recurrence, and bleeding rate in patients on anticoagulant therapy with warfarin compared to rivaroxaban in the Latin American population are limited in VTE.

**Methods:**

A retrospective cohort study with propensity score matching analysis was conducted in patients with pulmonary embolism and/or deep vein thrombosis anticoagulated with warfarin or rivaroxaban treated. Survival analysis was performed using a Kaplan-Meier curve for each of the intervention groups, and it was compared using a Log Rank test.

**Results:**

Of 2193 potentially eligible patients with a suspected diagnosis of VTE, 505 patients entered the analysis; of these, 285 subjects were managed with warfarin and 220 anticoagulated with rivaroxaban. Major bleeding at 12 months occurred in 2.7% (6/220) of patients treated with Rivaroxaban, compared to 10.2% (29/285) in the Warfarin group in the unmatched population (*p* = 0.001). In the matched population, bleeding at 12 months occurred in 2.9% (6/209) of patients on Rivaroxaban and in 11.0% (23/209) of patients on Warfarin (*p* = 0.001). The survival rates at 6 months were 97.1% for Rivaroxaban and 97.6% for Warfarin (*p* = 0.76). At 12 months, the survival rates were 94.7% for Rivaroxaban and 95.7% for Warfarin (*p* = 0.61).

**Conclusion:**

In the treatment of VTE, there is no differences on 6 and 12-month survival or a reduction in the occurrence of new thromboembolic events when comparing rivaroxaban to warfarin. However, a lower risk of major bleeding is observed at 12 months with Rivaroxaban.

## Introduction

Venous thromboembolism (VTE) is characterized by the obstruction of venous blood flow due to the formation of a thrombus, either in the lungs, known as pulmonary embolism (PE), or in the lower or upper extremities, referred to as deep vein thrombosis (DVT) [1]. VTE has an estimated annual risk of 100 cases per 100,000 individuals and an annual incidence of 0.1% in the United States [2]. In the case of DVT, the incidence is reported to range from 48 to 160 cases per 100,000 individuals per year worldwide [1–3]. In Latin America, particularly in Colombia, the prevalence of VTE is estimated to be 7% [4]. Given its pathophysiological characteristics and prevalence, anticoagulant therapy is considered essential to prevent thrombus extension and the formation of new clots within the venous system [1, 3, 5].

Vitamin K antagonists, such as warfarin, were once widely used for VTE treatment [6]. However, their use has declined due to a higher risk of severe bleeding episodes, drug interactions with other medications and food, and the need for regular monitoring and dose adjustments [4, 6]. In contrast, direct oral anticoagulants (DOACs) have emerged as a clinically relevant therapy, offering a superior profile of efficacy and effectiveness, as well as a lower risk of major bleeding and improved medication adherence [3, 5, 6].

Clinical trials have demonstrated a superior profile of efficacy and safety in terms of mortality and the risk of bleeding events for DOACs compared to warfarin [7, 8], including rivaroxaban, which can be initiated immediately after a VTE episode, facilitating its use, and enhancing clinical outcomes [9, 10]. However, there is limited published medical evidence from observational studies conducted in less controlled environments than clinical trials, which may reflect survival outcomes with greater applicability in clinical practice [4, 5, 7, 10]. Therefore, this study aims to evaluate 6- and 12-month survival in VTE patients treated with rivaroxaban and warfarin. Additionally, describe safety in terms of VTE recurrences and major bleeding at 12 months using a propensity score analysis.

## Methods

### Study design

An observational analytical study was conducted at a single center, using propensity score matching analysis, involving patients with VTE who were treated with warfarin or rivaroxaban at Clínica Universidad de La Sabana in Chía, Colombia, between August 2009 and January 2020.

### Inclusion criteria

Male and female individuals over 18 years of age who were prescribed warfarin or rivaroxaban for the treatment of DVT or PE were included. Patients with aortic aneurysms, suspected vascular lesions, non-traumatic aortic pathology, acute aortic syndrome, or pregnancy were excluded. Additionally, patients who received concurrent treatment with both warfarin and rivaroxaban and lacked medical history information, inaccessible radiology reports, or telephone follow-up were also excluded.

### Variables

VTE was defined as a confirmed diagnosis of PE through a positive radiological report of pulmonary artery computed tomography angiography, patients with a high probability report of ventilation/perfusion scintigraphy for PE, or a confirmed diagnosis of DVT through a positive report of lower extremity venous doppler performed by a radiologist.

The study included variables such as age, gender, comorbidities, symptoms, physical examination findings, D-dimer levels, troponin levels, echocardiogram results, chest X-ray, pulmonary artery computed tomography, venous doppler ultrasound of the lower extremities, need for mechanical ventilation, and admission to the intensive care unit. The primary outcome was measuring survival at 6 and 12 months through investigator-conducted telephone follow-up. Secondary outcomes included the occurrence of major bleeding at 12 months and the appearance of a new episode of VTE during follow-up, determined according to the previously mentioned VTE definition.

Major bleeding was defined based on the criteria set by the International Society on Thrombosis and Haemostasis (ISTH): clinically evident bleeding with a decrease in hemoglobin of > 2 g/dL within a 24-hour period, transfusion of 2 or more units of packed red blood cells, fatal bleeding, and bleeding occurring in any of the following critical sites: intracranial, intraspinal, intraocular, pericardial, intraarticular, intramuscular with compartment syndrome, and retroperitoneal [11].

Patients were followed from the initiation of anticoagulant therapy until the occurrence of the primary outcome, loss to follow-up, or completion of the study. Those patients who did not complete the 12-month follow-up were not excluded from the overall analysis but were censored in the survival analysis. Data collection from medical records was conducted by trained pairs of researchers. The researcher responsible for data analysis was not involved in data collection, and propensity score matching was used to reduce confounding bias.

### Sample size and bias

We determined the sample size based on the results of the study by Dennis et al. [12], which reported an 85% survival rate at 12 months post-hospitalization in PE patients. We considered a clinically significant difference of 15%, with an expected loss rate of 10%, a confidence level of 95%, and a power of 90%. Therefore, 208 patients were required for each study group, and patients were sequentially enrolled until the required number was reached.

### Statistical analysis

Data from medical records were collected using the electronic data capture software RedCap [13, 14] and then transferred to an Excel spreadsheet for analysis with IBM® SPSS® Statistics 25. An initial data review was conducted by variable to evaluate the percentage of data loss, which should not exceed 20%. If the threshold was exceeded, the variable was excluded from the analysis. Qualitative variables were summarized in terms of frequencies and percentages, while quantitative variables were expressed as means and standard deviations or medians and interquartile ranges, depending on their distribution. A bivariate analysis was performed using a Chi-squared test for quantitative variables, employing either Student’s t-test or Mann-Whitney U test based on the distribution. For qualitative variables, a statistically significant *p*-value < 0.05 was considered [15].

We selected independent variables with biological plausibility and statistically significant differences in both intervention groups for propensity score matching. A model was created using logistic regression with the intervention variable as the dependent variable and the predefined variables as the independent ones [15]. Using this model, the probability of each patient entering the two intervention groups (propensity score) was calculated. Subsequently, a 1:1 matching was conducted using the nearest neighbor matching without replacement technique [15]. A review was performed to ensure balance in the independent variables after matching by comparing standardized means, which were required to be < 5%. Finally, a survival analysis was carried out using a Kaplan-Meier curve for each intervention group, and a log-rank test was employed to establish whether a difference between them existed. A statistically significant value was considered when the test’s *p*-value was < 0.05.

## Results

### General characteristics

From an initial group of 2193 patients with suspected VTE who were potentially eligible, 505 patients were included in the analysis. Of these, 285 patients received Warfarin treatment, while 220 patients were anticoagulated with Rivaroxaban. In the unmatched population, the average age was 59.9 years (SD: 19.41), and 56.0% (283/505) were males. Similar data were observed in the matched group, with an average age of 60.8 (SD: 19.21) and 57.9% (242/418) males. In the unmatched population, dementia occurred in 3.6% (8/220) of patients on Rivaroxaban compared to 0.7% (2/285) in the Warfarin group (*p* = 0.02); inconsistent data were observed in the matched population. General characteristics are presented in Table 1.

**Table 1:** General characteristics

	No Match				Match			
	Total population *n*= 505	Rivaroxaban *n*= 220	Warfarin *n*= 285	*p* value	Total population *n*= 418	Rivaroxaban *n*= 209	Warfarin *n*= 209	*p* value
Age years, m(SD)	59.9 (19.41)	60.4 (18.94)	59.5 (19.79)	0.64	60.8 (19.21)	60 (18.59)	61.6 (19.82)	0.37
Male, n(%)	283 (56)	118 (53.6)	165 (57.9)	0.34	242 (57.9)	116 (55.5)	126 (60.3)	0.32
Comorbidities, n (%)								
Arterial hypertension	201 (39.8)	86 (39.1)	115 (40.4)	0.77	170 (40.7)	81 (38.8)	89 (42.6)	0.43
Congestive heart failure	28 (5.5)	12 (5.5)	16 (5.6)	0.94	24 (5.7)	12 (5.7)	12 (5.7)	1.00
Acute myocardial infarction	5 (1)	2 (0.9)	3 (1.1)	0.87	5 (1.2)	2 (1)	3 (1.4)	0.65
Atrial fibrillation	18 (3.6)	6 (2.7)	12 (4.2)	0.37	13 (3.1)	5 (2.4)	8 (3.8)	0.40
CE History	44 (8.7)	17 (7.7)	27 (9.5)	0.49	31 (7.4)	15 (7.2)	16 (7.7)	0.85
Dementia	10 (2)	8 (3.6)	2 (0.7)	0.02	6 (1.4)	4 (1.9)	2 (1)	0.41
COPD	45 (8.9)	18 (8.2)	27 (9.5)	0.61	35 (8.4)	17 (8.1)	18 (8.6)	0.86
Diabetes	57 (11.3)	23 (10.5)	34 (11.9)	0.60	52 (12.4)	23 (11)	29 (13.9)	0.37
Obesity	24 (4.8)	11 (5)	13 (4.6)	0.82	22 (5.3)	10 (4.8)	12 (5.7)	0.66
Cancer	39 (7.7)	19 (8.6)	20 (7)	0.50	35 (8.4)	19 (9.1)	16 (7.7)	0.60
Metástasis	13 (2.6)	7 (3.2)	6 (2.1)	0.45	13 (3.1)	7 (3.3)	6 (2.9)	0.78
Active cancer in the last year	18 (3.6)	10 (4.5)	8 (2.8)	0.30	18 (4.3)	10 (4.8)	8 (3.8)	0.63
Surgery in the last 4 weeks, n (%)	78 (15.4)	40 (18.2)	38 (13.3)	0.13	66 (15.8)	37 (17.7)	29 (13.9)	0.28
Knee replacement, n (%)	18 (3.6)	9 (4.1)	9 (3.2)	0.57	17 (4.1)	9 (4.3)	8 (3.8)	0.80
PE history, n (%)	23 (4.6)	6 (2.7)	17 (6)	0.08	12 (2.9)	6 (2.9)	6 (2.9)	1.00
DVT history, n (%)	96 (19)	30 (13.6)	66 (23.2)	0.01	63 (15.1)	30 (14.4)	33 (15.8)	0.68

*m* average, *SD* Standard deviation, *CE* Cerebrovascular event, *COPD* Chronic obstructive pulmonary disease, *PE* Pulmonary embolism, *DVT* Deep vein thrombosis

### Physical exam, laboratory test and diagnostic imaging

Chest pain was reported by 27.7% (61/220) of patients receiving Rivaroxaban, compared to 34.3% (98/285) of patients receiving Warfarin in the unmatched population (*p* = 0.11) Table 2. These results remained consistent in the matched population. Details of laboratory analyses and diagnostic imaging results are provided in Table 3.

**Table 2:** Physical exam: clinical manifestations and vitals signs

	No Match				Match			
	Total population *n*= 505	Rivaroxaban *n*= 220	Warfarin *n*= 285	*p* value	Total population *n*= 418	Rivaroxaban *n*= 209	Warfarin *n*= 209	*p* value
Dyspnoea, n(%)	149 (29.5)	63 (28.6)	86 (30.2)	0.71	122 (29.2)	58 (27.8)	64 (30.6)	0.52
Chest pain, n(%)	159 (31.5)	61 (27.7)	98 (34.4)	0.11	129 (30.9)	57 (27.3)	72 (34.4)	0.11
Hemoptisis, n(%)	21 (4.2)	6 (2.7)	15 (5.3)	0.16	18 (4.3)	6 (2.9)	12 (5.7)	0.15
Unilateral lower limb pain, n(%)	264 (52.3)	114 (51.8)	150 (52.6)	0.86	216 (51.7)	109 (52.2)	107 (51.2)	0.84
Unilateral lower limb edema, n(%)	264 (52.3)	113 (51.4)	151 (53)	0.72	216 (51.7)	108 (51.7)	108 (51.7)	1.00
Lower limb pain on palpation, n(%)	228 (45.1)	97 (44.1)	131 (46)	0.67	188 (45)	92 (44)	96 (45.9)	0.69
Non-varicose collateral veins, n(%)	8 (1.6)	3 (1.4)	5 (1.8)	0.73	5 (1.2)	2 (1)	3 (1.4)	0.65
VD in the lower limbs, n(%)	21 (4.2)	7 (3.2)	14 (4.9)	0.33	16 (3.8)	7 (3.3)	9 (4.3)	0.61
DD of lower limbs >3cm, n(%)	84 (16.6)	32 (14.5)	52 (18.2)	0.27	70 (16.7)	32 (15.3)	38 (18.2)	0.43
Temperature, m(SD)	36.2 (3.21)	36.3 (2.49)	36.1 (3.66)	0.64	36.2 (3.06)	36.2 (2.56)	36.2 (3.49)	0.75
Systolic blood pressure, m(SD)	126.6 (18.88)	127.7 (19.02)	125.7 (18.76)	0.24	127.2 (18.76)	127.3 (18.57)	127.1 (18.98)	0.93
Diastolic blood pressure, m(SD)	75.6 (11.85)	75.5 (11.91)	75.7 (11.81)	0.83	75.8 (11.51)	75.4 (11.62)	76.2 (11.42)	0.48
Heart rate, m(SD)	85.8 (16.16)	85.3 (15.67)	86.2 (16.54)	0.52	85.9 (15.83)	85.4 (15.95)	86.4 (15.72)	0.51
Oxygen saturation, m(SD)	91.1 (7.68)	90.7 (8.13)	91.4 (7.32)	0.35	91 (6.95)	90.7 (8.32)	91.3 (5.27)	0.36

*VD* Venous distension, *DD* Difference in diameter, *m* average, *SD* Standard deviation

**Table 3:** Laboratory tests and diagnostic images

	No Match				Match			
	Total population *n*= 505	Rivaroxaban *n*= 220	Warfarin *n*= 285	*p* value	Total population *n*= 418	Rivaroxaban *n*= 209	Warfarin *n*= 209	*p* value
D-dimer, m(SD)	4.5 (3.37)	4.3 (3.25)	4.6 (3.48)	0.34	4.6 (3.38)	4.4 (3.19)	4.9 (3.59)	0.16
Troponin, m(SD)	0.2 (0.62)	0.2 (0.71)	0.2 (0.53)	0.92	0.2 (0.69)	0.2 (0.74)	0.2 (0.63)	0.63
chest x-ray; n (%)	207 (41)	89 (40.5)	118 (41.4)	0.83	161 (38.5)	81 (38.8)	80 (38.3)	0.92
Hilar artery amputation, n (%)	5 (1)	2 (0.9)	3 (1.1)	0.87	4 (1)	2 (1)	2 (1)	1.00
Consolidation due to PI, n (%)	14 (100)	6 (100)	8 (100)	0.96	12 (100)	6 (100)	6 (100)	1.00
Consolidation without to PI, n (%)	32 (6.3)	16 (7.3)	16 (5.6)	0.45	26 (6.2)	14 (6.7)	12 (5.7)	0.69
Pulmonary edema, n (%)	12 (2.4)	4 (1.8)	8 (2.8)	0.47	11 (2.6)	4 (1.9)	7 (3.3)	0.36
Echocardiogram, n (%)	150 (29.7)	73 (33.2)	77 (27)	0.13	123 (29.4)	65 (31.1)	58 (27.8)	0.45
TAPSE, n (%)	1.8 (0.41)	1.8 (0.39)	1.8 (0.44)	0.77	1.8 (0.43)	1.8 (0.41)	1.8 (0.46)	0.20
Pressure overload of right cavities, n (%)	18 (3.6)	10 (4.5)	8 (2.8)	0.30	15 (3.6)	7 (3.3)	8 (3.8)	0.79
paradoxical movement, n (%)	2 (0.4)	1 (0.5)	1 (0.4)	0.85	2 (0.5)	1 (0.5)	1 (0.5)	1.00
Deviation of the interventricular septum, n (%)	3 (0.6)	2 (0.9)	1 (0.4)	0.42	3 (0.7)	2 (1)	1 (0.5)	0.56
DVT diagnosis, n (%)	265 (52.5)	111 (50.5)	154 (54)	0.27	219 (52.4)	108 (51.7)	111 (53.1)	0.48
PE with DVT diagnosis, n (%)	54 (10.7)	20 (9.1)	34 (11.9)	0.16	40 (9.6)	17 (8.1)	23 (11)	0.34
PE diagnosis, n (%)	186 (36.8)	89 (40.5)	97 (34)	0.24	159 (38)	84 (40.2)	75 (35.9)	0.30

*m* average, *SD* standard deviation, *PI* Pulmonary infarction, *TAPSE* Tricuspid Annular Plane Systolic Excursion, *PE* Pulmonary embolism, *DVT* Deep vein thrombosis

### Safety: major bleeding

In terms of clinical outcomes, major bleeding at 12 months occurred in 2.7% (6/220) of patients treated with Rivaroxaban, compared to 10.2% (29/285) in the Warfarin group in the unmatched population (*p* = 0.001) Table 4. In the matched population, bleeding at 12 months occurred in 2.9% (6/209) of patients on Rivaroxaban and in 11.0% (23/209) of patients on Warfarin (*p* = 0.001).

**Table 4:** Treatment and clinical outcomes

	No Match				Match			
	Total population *n*= 505	Rivaroxaban *n*= 220	Warfarin *n*= 285	*p* value	Total population *n*= 418	Rivaroxaban *n*= 209	Warfarin *n*= 209	*p* value
Admission to ICU, n(%)	58 (11.5)	21 (9.5)	37 (13)	0.23	37 (8.9)	20 (9.6)	17 (8.1)	0.61
Need for IMV, n(%)	13 (2.6)	5 (2.3)	8 (2.8)	0.71	8 (1.9)	4 (1.9)	4 (1.9)	1.00
Need for thrombolysis, n(%)	15 (3)	4 (1.8)	11 (3.9)	0.18	6 (1.4)	4 (1.9)	2 (1)	0.41
Hospital stay days, m(SD)	8.5 (14.33)	7.4 (12.82)	9.3 (15.37)	0.13	7.8 (10.95)	7.4 (13.08)	8.2 (8.32)	0.50
Initial treatment with enoxaparin, n(%)	449 (88.9)	176 (80)	273 (95.8)	<0.01	367 (87.8)	166 (79.4)	201 (96.2)	<0.01
Major bleeding at 12 months, n(%)	35 (6.9)	6 (2.7)	29 (10.2)	0.01	29 (6.9)	6 (2.9)	23 (11)	0.01
New episode of VTE	30 (5.9)	12 (5.5)	18 (6.3)	0.68	27 (6.5)	12 (5.7)	15 (7.2)	0.55

*m* average, *SD* Standard deviation, *ICU* Intensive critical unit, *IMV* Invasive mechanical ventilation, *VTE* Venous thromboembolism

### Survival at 6 and 12 months

**Fig. 1:**
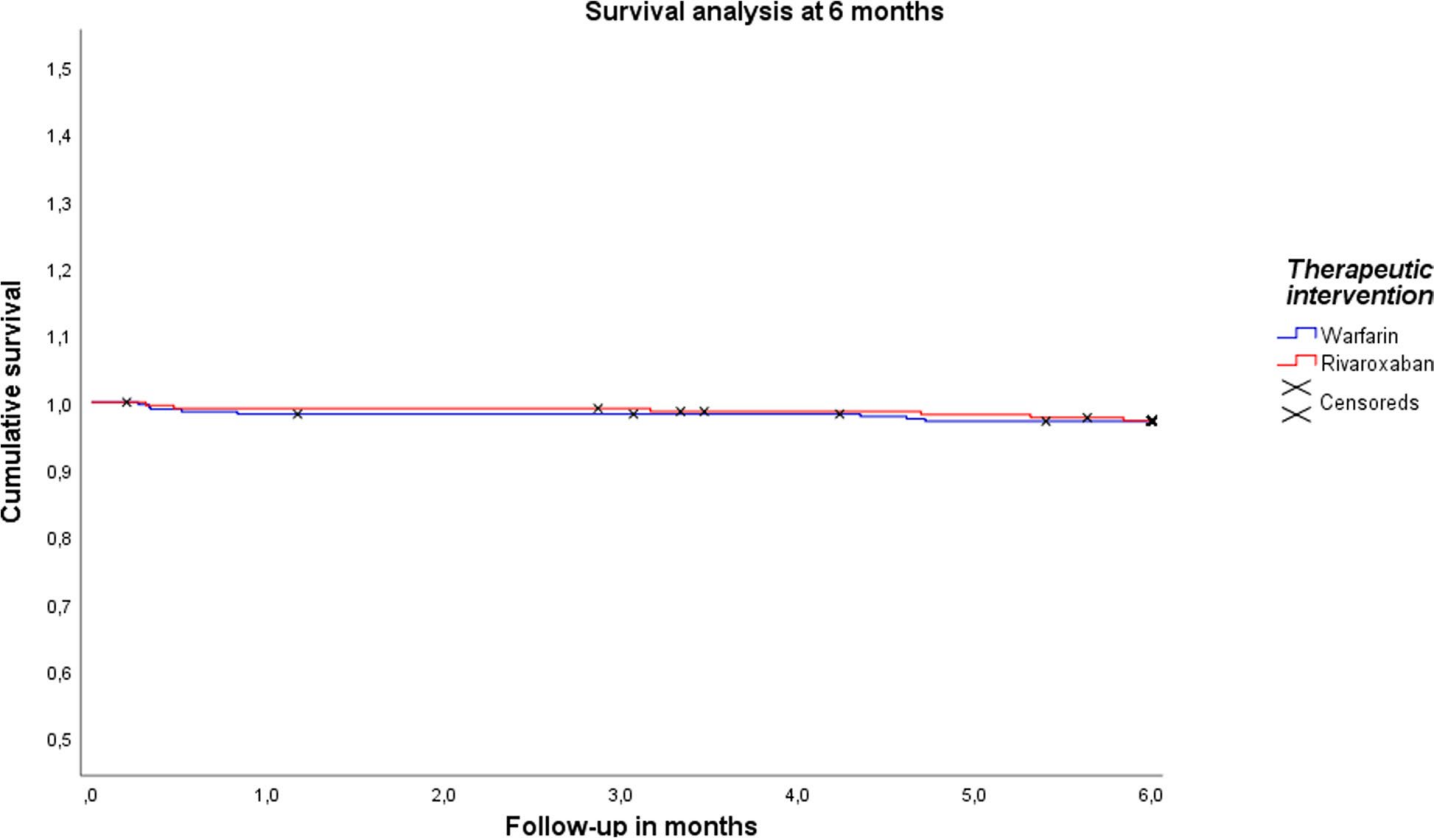
Survival analysis at 6 months.  Notes : A Kaplan-Meier survival analysis was performed for the warfarin vs. rivaroxaban groups at 6 months, and group comparisons were performed using the log-rank test (*p* = 0.76)

**Fig. 2:**
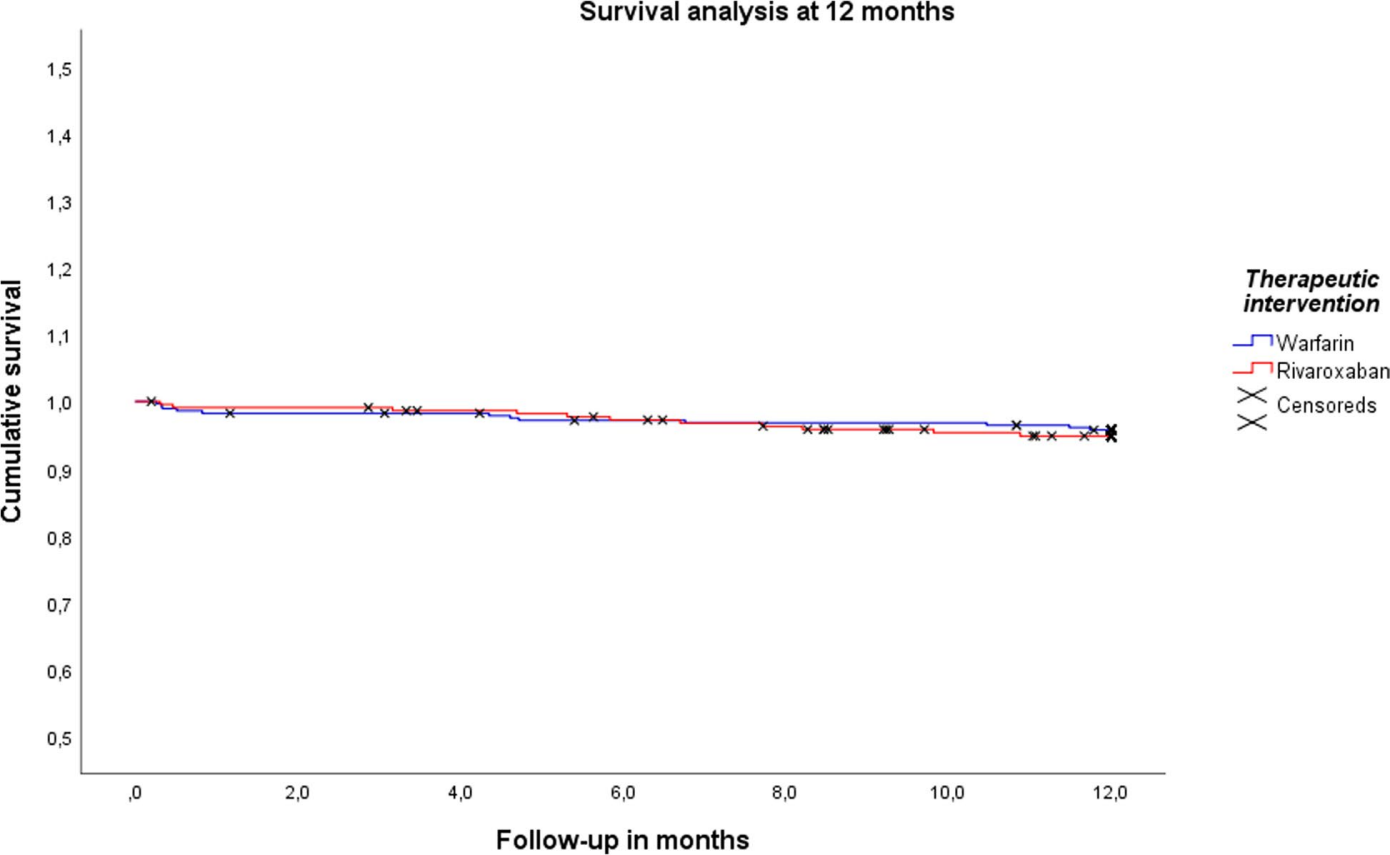
Survival analysis at 12 months.  Notes : A Kaplan-Meier survival analysis was performed for the warfarin vs. rivaroxaban groups at 12 months, and group comparisons were performed using the log-rank test (*p* = 0.61)

The survival rates at 6 months were 97.1% for Rivaroxaban and 97.6% for Warfarin (*p* = 0.76) (see Fig. 1). At 12 months, the survival rates were 94.7% for Rivaroxaban and 95.7% for Warfarin (*p* = 0.61) (see Fig. 2).

## Discussion

This study revealed that patients with VTE under treatment with rivaroxaban versus warfarin at 6 and 12 months did not show differences in survival or the occurrence of new VTE episodes. However, a lower risk of major bleeding at 12 months was identified with the administration of Rivaroxaban. A higher frequency of dementia was observed in the rivaroxaban treatment group compared to the group that received warfarin. The results of our study are consistent with the existing literature regarding the bleeding rate of DOACs and coumarins [16–20].

Coleman et al. [16] conducted a propensity score matching analysis to compare the effectiveness and safety of rivaroxaban versus warfarin in patients with unprovoked VTE. The results demonstrated a reduction in bleeding events at 3, 6, and 12 months, as well as a decrease in the occurrence of new thromboembolic events during the same time periods. To date, there is no medical literature that examines survival at 6 and 12 months in patients diagnosed with VTE and anticoagulated with rivaroxaban compared to warfarin, suggesting that our findings, in conjunction with those presented by Coleman, indicate a potential decrease in the risk of major bleeding and VTE recurrence.

Larsen et al. [17] conducted a comparative analysis of the efficacy and safety of rivaroxaban versus warfarin, revealing a reduction in the recurrence of thromboembolic events with rivaroxaban (HR: 0.69, 95% CI: 0.55–0.87). No significant differences were observed in the risk of bleeding (HR: 1.18; 0.69–2.04) or all-cause mortality (HR: 1.23; 0.91–1.67) between both anticoagulants. Van der Hulle et al. [18] compared the use of warfarin with DOACs (rivaroxaban, dabigatran, apixaban, and edoxaban) in VTE patients. In this comparison of five randomized clinical trials, a significant decrease was observed in the occurrence of major bleeding (RR: 0.60; 95% CI 0.41–0.88), nonfatal bleeding (RR: 0.38; 95% CI 0.23–0.62), clinically relevant non-major bleeding (RR: 0.76; 95% CI 0.58–0.99), nonfatal intracranial bleeding (RR 0.39; 95% CI 0.16–0.94), and fatal bleeding (RR: 0.36; 95% CI 0.15–0.87) in the group of patients treated with DOACs. Our results indicate a lower risk of major bleeding with the use of rivaroxaban, although we did not analyze patients with non-major or minor bleeding events.

The EINSTEIN clinical trial did not demonstrate a higher rate of VTE recurrence (HR: 0.68; 95% CI: 0.44–1.04) in patients treated with rivaroxaban compared to those treated with warfarin [19]. Similarly, the EINSTEIN-PE study revealed a decrease in the risk of major bleeding with the use of rivaroxaban compared to standard therapy (HR: 0.49; 95% CI: 0.31–0.79), albeit with an increased risk of new episodes of VTE (HR: 1.12; 95% CI: 0.75–1.68) [20]. Despite the controversy in clinical outcomes reported in the literature, our data are consistent with a reduction in the risk of bleeding associated with the use of rivaroxaban compared to coumarins in patients diagnosed with VTE.

A strong causal association between the use of Rivaroxaban and the development of dementia has not been established [21, 22]. Lin et al. [21]. conducted a study comparing the safety and effectiveness of DOACs (apixaban, dabigatran, and Rivaroxaban) in a group of 1,160,462 patients with atrial fibrillation. In this study, the average age of patients was 77.4 years, and the prevalence of dementia was 7.9%. They found that, compared to apixaban users, Rivaroxaban users had higher rates of major bleeding, ischemic stroke, and mortality [21].

The relationship between the use of anticoagulants like Rivaroxaban and dementia may be associated with the risk of significant intracranial bleeding, which, in turn, could lead to a negative impact on cognitive function, especially in elderly patients [21–24]. In our results, a higher frequency of dementia was observed in patients using Rivaroxaban in the unmatched population. However, these results did not reach statistical significance when a propensity score analysis was performed, suggesting a weak association between the use of Rivaroxaban and the development of dementia. Further prospective observational studies or clinical trials are needed to better describe and understand this association.

### Limitations

Among the limitations of the study, the exclusive inclusion of patients from a single hospital stands out, which limits the generalization of the results to other populations. The quality of information in an observational study relies on the contents of the medical records and the competence of the research team in extracting this data. However, the researchers are adequately medically trained to ensure precise data interpretation and collection.

The study was conducted in a highly complex institution, which carried a substantial risk of selection and prevalence bias. This is because patients with severe thromboembolic diseases might pass away prior to receiving oral anticoagulation therapy, which would exclude them from the study. This could, in turn, increase the inclusion of subjects with milder diseases, significantly affecting the mortality rate in a biased manner. Nonetheless, the study boasts a sufficient sample size for comparative analysis. Another limitation was the lack of monitoring of prothrombin time and international normalized ratio (PT/INR) values for patients in the warfarin group, which is a crucial covariate for measuring the risk of bleeding. Additionally, deaths occurring during enoxaparin bridging therapy in patients on warfarin were not considered, leading to an immortal time bias. To mitigate this bias, the start date of follow-up was chosen as the commencement of anticoagulant treatment.

Our findings suggest the possibility of future studies assessing the economic implications of rivaroxaban compared to warfarin in VTE patients in the Colombian population.

## Conclusion

In the treatment of VTE, there is no differences on 6 and 12-month survival or a reduction in the occurrence of new thromboembolic events when comparing rivaroxaban to warfarin. However, a lower risk of major bleeding is observed at 12 months with Rivaroxaban compared to warfarin.

## Data Availability

The datasets generated during and/or analyzed during the current study are available from the corresponding author on reasonable request.
